# Dictatorial

**Published:** 1882-04

**Authors:** 


### EDITORIAL, ETC.

---

*Dictatorial.*—When, at the Southern Dental Association in July last, the writer of this, put into words of his own, the thoughts of Prof. Winder, of the Baltimore College of Dental Surgery, on the subject of Dental Education, he little thought that they would receive the hearty endorsement they have in so many quarters. That these resolutions were founded on principles of truth and common sense was felt, but in the advanced ground then taken it was apprehended that only slowly would the truths involved be sanctioned. From many quarters comes recognition of the sound doctrine contained in the resolutions passed unanimously by the Southern Dental Association, and of approval of these "resolutions." That the time will come when all must recognize the justice of the spirit of these, we are quite sure; just as we are sure that those who are at all observant must know that some men learn faster than others. The quality of the mind is so different in different students that arbitrary rules have long ago been thrown aside by intelligent teachers and the individuality of the student recognized. Were the class of a dental college to remain as a whole, after drill, as soldiers, the drill *en masse* would be proper, but as its integrity is broken at graduation and each man is free to follow his individuality, it is proper to recognize this and allow for it during the developmental stage—indeed this is *the* time for its full recognition. We have in mind two most excellent and successful practitioners, standing high as operators and men of worth, one of whom learned more of dentistry, theoretically and practically in three months than the other did in two years, yet each had equally good opportunities as students. And this illustration must be verified by every observant teacher.

In view of all this, it does seem, with all respect to that respectable body of representative men, the American Dental Association, that the resolution passed in 1880 has an air of dictation not authorized by the powers of that body. To compel any man to remain in the ranks with students, when he has mastered the studies set for the class, is a hardship no college or association has a right to inflict. It is sheer injustice. It is argued that the hardship seldom occurs in fact; but this does not relieve occasional fact of its unjust character. Dental colleges rather encourage young men to enter their classes; to unnecessarily hinder their departure, *if fit*, is wrong, and yet this wrong is what the American Dental Association urges. As was said not long ago by one of the first scientists and teachers of the age, we have no *right* to handicap the intelligent student with the weight of the dull and sluggish.

We append a few quotations from *Items of Interest* on this subject, and publish together the resolutions of the bodies representing largely the colleges of the North and East, and those of the Southern Dental Association, as a contrast.

[From Items of Interest.]

"A title from a college, whether of dentistry, medicine or law, does not signify what the college has made the recipient, but what their examination finds him to possess. What matters it *how* he comes to be what he is, if only his attainments honor the title as much as the title honors him?

"With the certificate of qualification called a diploma, given by the gentlemen representing a dental college, the recipient of their honors is no better qualified to practice dentistry after receiving his decrees than before. It simply represents the opinion of these men that he is worthy of the confidence of the community as a dentist.

"In charity, we might suppose we misinterpret the language cf those composing the American Dental Association. At home they are modest. It seems impossible that these dentists who in their offices are simply our fellows, should, when abroad, assume such a self-constituted, self-conceited and unwarrantable position. Yet they make their assumption unequivocal by the following additional imperious declaration: 'This association claims the right to exercise a general supervision over the whole subject of dental education.' The law-makers of the several states give our colleges vested rights over dental education; but who gives this company of dentists, 'a general supervision,' not only over dental education but over dental colleges too?"

[Resolutions American Dental Association.]

"*Resolved*, That in order to secure representation in this association, dental colleges must, subsequent to October 1st, 1881, require all students entering therein to take two full courses of lectures previous to coming forward for examination and graduation, and must also state these conditions in their next annual announcement."

"*Resolved*, That the Southern Dental Association place itself on record as indorsing the principles of education as enunciated by the University of Virginia at its foundation, and of the Johns Hopkins University, cf Baltimore, Maryland, of conferring degrees according to merit and previous acquirements; and that we indorse heartily the doctrine that a diploma from a dental college should be awarded to merit, and merit alone, irrespective of the number of years of previous study; and, further, that inasmuch as knowledge is equally valuable whenever and wherever obtained, this association indorses, first the doctrine of demanding the highest standard of attainments compatible with the present onward march of dentistry, on the part of applicants for degrees; and, second, the most liberal spirit on the part of those granting diplomas as to the manner in which said knowledge is obtained; that it is unjust and irrational on the part of any teacher to demand of a pupil the attendance on any prescribed and lengthy course of lectures, if it is proved satifactorily that the requisite knowledge has been already attained; and that we believe that, in justice to a student of dentistry, and as well as of any other study, the privilege of graduating whenever pronounced fit, is the true principle." H.

